# PROCESSES OF EXCLUSION AND NEW RURALITY: COMMUNITY CHANGE IN THE LIVES OF RURAL OLDER PEOPLE

**DOI:** 10.1093/geroni/igz038.1475

**Published:** 2019-11-08

**Authors:** Sinead Keogh, Kieran Walsh

**Affiliations:** 1 National University of Ireland Galway, Galway, Ireland, Ireland; 2 Irish Centre for Social Gerontology, Galway, Galway, Ireland

## Abstract

Rural settings are sites of rapid change. Now sharing many of the processes that characterise their urban neighbourhood counterparts, older people’s rural communities, even those in remote locations, are being altered by forces driven by gentrification and population churn. While the potential for displacement is apparent, the extent to which older people respond to these processes is not well understood. The degree to which these shifting contexts produce new exclusionary mechanisms for older people to contend with and new opportunities for them to exploit has yet to be sufficiently explored. This paper aims to address the intersection of exclusion and community change in the production of a new rurality for older people. The analysis will 1) present an overview of the relevant international literature, and 2) highlight the current and emerging exclusionary processes that are impacting on the lives of older people using data from individual narratives and time-use diaries.

